# Comparison of viral infection in healthcare-associated pneumonia (HCAP) and community-acquired pneumonia (CAP)

**DOI:** 10.1371/journal.pone.0192893

**Published:** 2018-02-15

**Authors:** Eun Sun Kim, Kyoung Un Park, Sang Hoon Lee, Yeon Joo Lee, Jong Sun Park, Young-Jae Cho, Ho Il Yoon, Choon-Taek Lee, Jae Ho Lee

**Affiliations:** 1 Department of Internal Medicine, Seoul National University College of Medicine, Seoul, Republic of Korea; 2 Division of Pulmonary and Critical Care Medicine, Department of Internal Medicine, Seoul National University Bundang Hospital, Seongnam-si, Gyeonggi-do, Republic of Korea; 3 Department of Laboratory Medicine, Seoul National University Bundang Hospital, Seongnam-si, Gyeonggi-do, Republic of Korea; University of Edinburgh, UNITED KINGDOM

## Abstract

**Background:**

Although viruses are known to be the second most common etiological factor in community-acquired pneumonia (CAP), the respiratory viral profile of the patients with healthcare-associated pneumonia (HCAP) has not yet been elucidated. We investigated the prevalence and the clinical impact of respiratory virus infection in adult patients with HCAP.

**Methods:**

Patients admitted with HCAP or CAP, between January and December 2016, to a tertiary referral hospital in Korea, were prospectively enrolled, and virus identification was performed using reverse-transcription polymerase chain reaction (RT-PCR).

**Results:**

Among 452 enrolled patients (224 with HCAP, 228 with CAP), samples for respiratory viruses were collected from sputum or endotracheal aspirate in 430 (95.1%) patients and from nasopharyngeal specimens in 22 (4.9%) patients. Eighty-seven (19.2%) patients had a viral infection, and the proportion of those with viral infection was significantly lower in the HCAP than in the CAP group (13.8% *vs* 24.6%, p = 0.004). In both the HCAP and CAP groups, influenza A was the most common respiratory virus, followed by entero-rhinovirus. The seasonal distributions of respiratory viruses were also similar in both groups. In the HCAP group, the viral infection resulted in a similar length of hospital stay and in-hospital mortality as viral–bacterial coinfection and bacterial infection, and the CAP group showed similar results.

**Conclusions:**

The prevalence of viral infection in patients with HCAP was lower than that in patients with CAP, and resulted in a similar prognosis as viral–bacterial coinfection or bacterial infection.

## Introduction

Patients increasingly receive treatment at facilities other than hospitals, including long-term health care facilities, due to the rapidly increasing aged population, who more often have chronic diseases [1, 2] Pneumonia occurring prior to hospital admission in patients who have had recent contact with health systems is termed healthcare-associated pneumonia (HCAP) [3–5]. These patients are believed to be at increased risk for infection with multidrug-resistant (MDR) organisms [3, 4], and is of particular concern in HCAP patients. However, recent studies have indicated that many patients defined as having HCAP were not infected with MDR pathogens [6–10]. Even after excluding MDR pathogens, substantial differences in the etiological profile between community-acquired pneumonia (CAP) and HCAP groups have been noted in recent studies [11, 12], indicating that HCAP settings might present unique risks of pneumonia.

The incidence of viral pneumonia has increased during the past decade [13]. In part, this apparent increase reflects improved diagnostic techniques, including polymerase chain reaction (PCR) [13]. Over the last decade, several studies have used PCR to establish the importance of viruses in the etiology of CAP, and have consistently demonstrated viruses to be the second most common etiological factor, accounting for 13–50% of diagnosed cases [7, 11, 13–21]. In a recent systematic review and meta-analysis of viral CAP, no significant association between viral infection and increased mortality was observed, but mortality was increased in patients with a bacterial–viral coinfection [22]. However, its clinical implications are debatable, because most studies performed PCR using nasopharyngeal or oropharyngeal swabs [22], which might yield a negative result in patients with lower respiratory tract viral infection [11, 23].

Unlike CAP, the viral profile and the clinical implications thereof in HCAP are not well studied. Therefore, we performed this prospective study 1) to investigate the role of viral infection in patients with HCAP and CAP, using all available adequate respiratory specimens from our institution, and 2) to identify the seasonal variation in both groups of patients.

## Materials and methods

### Study design and patients

This study was conducted at a tertiary referral hospital in Republic of Korea. All adult patients, who were admitted to the hospital with HCAP or CAP from January 1 to December 31, 2016, were prospectively recruited after providing written informed consent. Patients were excluded from the study if they had pneumonia related to witnessed aspiration during eating or vomiting, obstructive pneumonia related to airway obstruction by a tumor, hospital-acquired (HAP), or ventilator-associated pneumonia (VAP). Patients who did not undergo any microbiological studies before antibiotic administration, who received any form of antibiotics for more than 24 hrs prior to our hospital or who refused to participate in this study were also excluded. If a patient had multiple pneumonia episodes within the study period, only the first pneumonia episode was recorded. The study was approved by the Institutional Review Board and Ethics Committee of Seoul National University Bundang Hospital (SNUBH) (IRB No. B-1511-324-306) and was conducted in compliance with the Declaration of Helsinki.

### Definitions

Pneumonia was defined as the presence of new infiltrates on chest X-rays along with other suggestive signs and symptoms: cough, sputum, fever, chills, dyspnea, pleuritic chest pain, disturbance of consciousness, and crackles [3, 24]. The patients with HCAP had to fulfill any of the following: received intravenous therapy at home; received wound care or nursing care through a health care agency; or had intravenous medical therapy in the 30 days before pneumonia; or attended a hemodialysis clinic; or received intravenous chemotherapy in the 30 days before pneumonia; or admitted to a hospital for 2 or more days in the 90 days before pneumonia; or resided in a nursing home or a long-term care facility. Patients were classified into the CAP if they did not fit the criteria for HCAP.

### Microbiological studies

Microbiological samples were collected within 48 hr after diagnosis of pneumonia. Microbiological studies included 2 or 3 sets of blood cultures and sputum or endotracheal aspirates for Gram staining and culturing. Urinary antigen tests for *Streptococcus pneumoniae* (Binax Inc., Portland, ME) and PCR tests for *Mycoplasma pneumoniae* (in-house developed nucleic acid sequence based amplification) were performed when indicated by the attending physician. Bronchoalveolar lavage (BAL) fluid or thoracentesis was conducted if indicated for the diagnosis. All patients were tested for respiratory virus by multiplex reverse-transcription PCR (RT-PCR), using all available respiratory specimens. If the patients had no sputum, RT-PCR was conducted on a nasopharyngeal specimen. Respiratory virus multiplex RT-PCR was performed according to the xTAG respiratory viral panel (RVP) assay product insert instructions (Luminex Molecular Diagnostics, Toronto, Canada). We considered that pneumonia was caused by a specific virus if respiratory viruses were detected in acceptable respiratory samples by RT-PCR [25]. Respiratory samples were considered acceptable when more than 25 polymorphonuclear cells and fewer than 10 squamous squamous epithelial cells were observed under low-power magnification were observed under low-power magnification [26–30].

### Statistical analysis

Unless otherwise specified, results are expressed as mean (standard deviation, SD) or median (range) for continuous variables and as percentage for categorical variables. Student’s *t*-test was used to compare continuous variables; chi-square or Fisher’s exact tests were used to compare categorical variables. Variables with p < 0.1 in univariate analysis were entered into a multivariate logistic regression analysis to identify independent predictors of mortality. Unless otherwise noted, all tests were 2-sided and performed at the 0.05 significance level. Analyses were performed using SPSS 20.0 (IBM Corp., Armonk, NY, USA).

## Results

### Baseline characteristics

**Fig 1:**
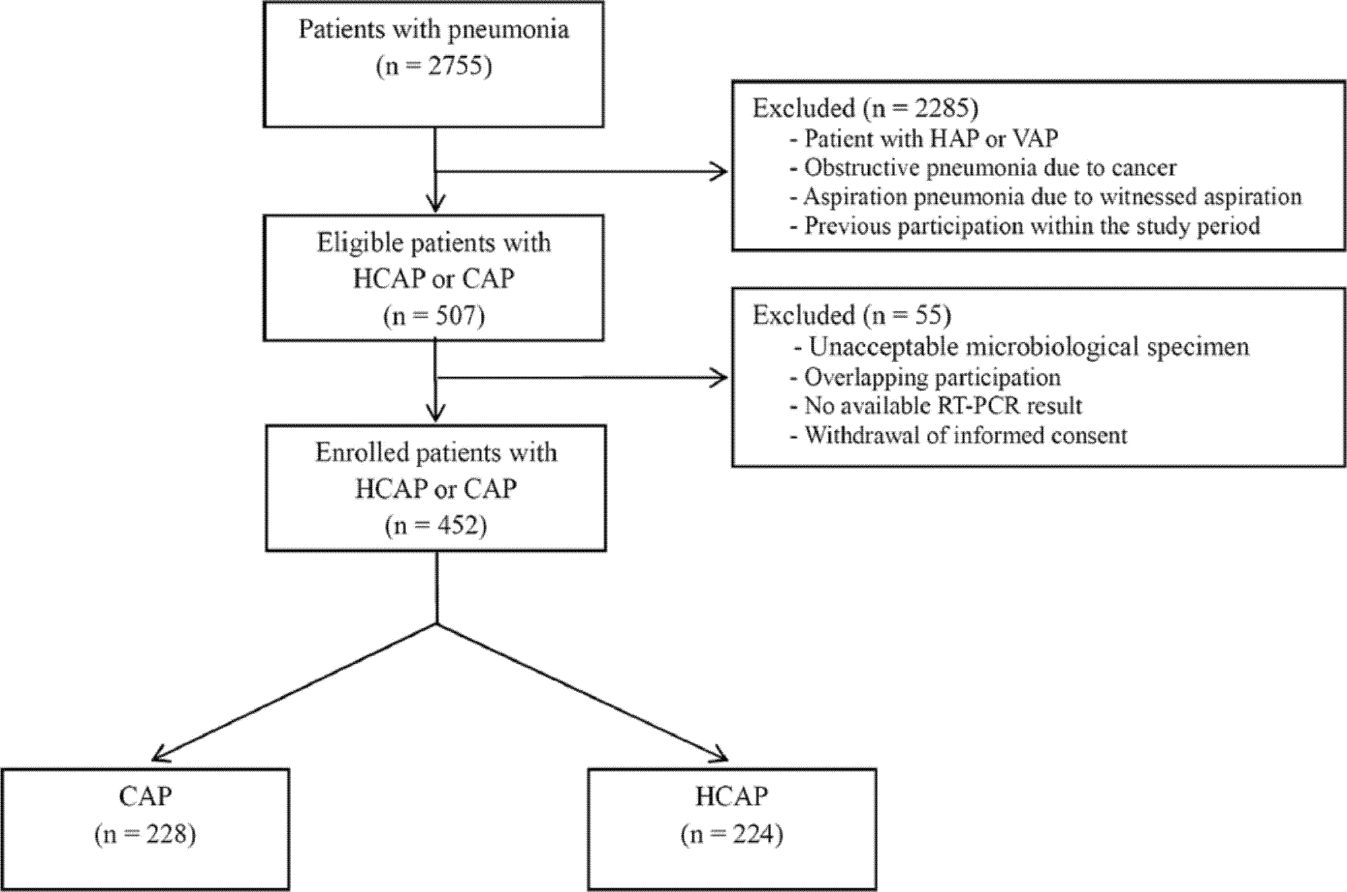
Flow chart of patient enrollment into the study. **Abbreviations:** CAP, community-acquired pneumonia; HAP, hospital-acquired pneumonia; HCAP, healthcare-associated pneumonia; VAP, ventilator-associated pneumonia; RT-PCR, reverse-transcription polymerase chain reaction.

Among 2755 patients who visited the emergency department due to pneumonia during the 1-year study period, 507 patients were diagnosed with HCAP or CAP. Fifty-five patients were excluded from the study because the microbiological specimens obtained prior to antibiotic administration were unacceptable (n = 37), or due to overlapping participation (n = 9); absence of RT-PCR results (n = 6); or withdrawal of informed consent (n = 3) (Fig 1). The baseline characteristics of 452 patients (HCAP: 224 patients; CAP: 228 patients) are shown in Table 1. Conventional microbiological studies, including blood culturing and sputum or endotracheal aspirate Gram staining and culturing, were conducted in all enrolled patients before or just after administration of antibiotics (within 1 hr). BAL was conducted in 67 (14.8%) patients and additional diagnostic thoracentesis was performed in 19 (4.2%) patients. The proportions of patients who underwent BAL and thoracentesis were similar in the CAP and HCAP groups (BAL, 12.3% *vs* 17.4%, p = 0.125; thoracentesis, 3.5% *vs* 4.9%, p = 0.458). Pathogens related to pneumonia were identified in 235 (52.0%) patients. Among them, 107 (23.7%) patients had bacterial pneumonia, while 87 (19.2%) patients had viral, and 41 (9.1%) patients had viral–bacterial coinfection. (S1 Table).

**Table 1:** Baseline characteristics of enrolled patients.

	Total (n = 452)	HCAP (n = 224)	CAP (n = 228)	*P* value
Male sex	279 (62.7)	142 (63.4)	137 (60.1)	0.470
Age, years	70.5 (15.0)	72.4 (11.5)	68.6 (17.6)	0.006
BMI, kg/m^2^	21.8 (4.2)	21.1 (4.1)	22.4 (4.3)	0.002
Ever-smoker	120 (26.5)	49 (21.9)	71 (31.1)	0.026
Alcoholism	52 (11.5)	16 (7.1)	36 (15.8)	0.004
In the army	7 (1.5)	0 (0.0)	7 (3.1)	0.015
ECOG ≥ 3	187 (41.4)	123 (54.9)	64 (28.1)	< 0.001
Comorbidities				
Diabetes mellitus (any type)	129 (28.5)	73 (32.6)	56 (24.6)	0.063
Hypertension	214 (47.3)	111 (49.6)	103 (45.2)	0.351
Chronic liver disease	14 (3.1)	8 (3.6)	6 (2.6)	0.564
Chronic kidney disease	35 (7.7)	21 (9.4)	14 (6.1)	0.198
Ischemic heart disease	74 (16.4)	46 (20.5)	28 (12.3)	0.018
Congestive heart failure	18 (4.0)	10 (4.5)	8 (3.5)	0.603
Chronic lung disease	109 (24.1)	35 (15.6)	74 (32.5)	< 0.001
Bronchiectasis	25 (5.5)	5 (2.2)	10 (0.4)	0.002
COPD	62 (13.7)	23 (10.3)	39 (17.1)	0.035
Bronchial asthma	38 (8.4)	6 (2.7)	32 (14.0)	< 0.001
Interstitial lung disease	7 (1.5)	3 (1.3)	4 (1.8)	1.000
Solid cancer	85 (18.8)	66 (29.5)	19 (8.3)	< 0.001
Hematologic malignancy	47 (10.4)	33 (14.7)	14 (6.1)	0.003
Aspiration pneumonia	85 (18.8)	62 (27.7)	23 (10.1)	< 0.001
Pneumonia severity index	105.6 (36.3)	123.0 (29.8)	88.3 (34.0)	< 0.001
CURB-65 ≥ 2	234 (51.8)	137 (61.2)	97 (42.5)	< 0.001
CURB-65 ≥ 3	81 (17.9)	50 (22.3)	31 (13.6)	0.016
qSOFA ≥ 2	57 (12.6)	46 (20.5)	11 (4.8)	< 0.001
Direct sub-ICU admission	35 (7.7)	24 (10.7)	11 (4.8)	0.019
Direct ICU admission	27 (6.0)	16 (7.1)	11 (4.8)	0.298
Initial Sepsis	6 (1.3)	4 (1.8)	2 (0.9)	0.399
Initial septic shock	11 (2.4)	8 (3.6)	3 (1.3)	0.120
Initial laboratory findings				
White blood cells /mm^3^	11.8 (8.3)	11.1 (6.5)	12.6 (9.8)	0.058
Hematocrit, %	36.1 (6.1)	33.9 (6.4)	38.3 (5.1)	< 0.001
Platelets, 10^3^/mm^3^	222.6 (110.9)	218.4 (117.7)	216.7 (103.9)	0.425
BUN, mg/dL	24.5 (19.7)	27.3 (20.9)	21.8 (18.0)	0.003
Creatinine, mg/dL	1.3 (1.3)	1.3 (1.3)	1.2 (1.3)	0.529
CRP, mg/dL	11.8 (7.8)	12.1 (7.6)	11.6 (7.9)	0.535
Procalcitonin (n = 205), ng/mL	0.5 (0.0–32.1)	1.5 (0.1–32.1)	0.5 (0.0–13.3)	0.014
Pro BNP (n = 203), pg/mL	660.7 (10.5–35000)	779.7 (143.5–22663.4)	400.3 (10.5–35000)	0.315
Lactic acid (n = 302)	1.4 (0.2–5.9)	1.1 (0.2–5.9)	1.5 (0.6–4.1)	0.019
D-dimer (n = 323), μg/mL	2.0 (0.4–20.0)	2.4 (0.4–20.0)	1.7 (0.4–20.0)	0.005

**Note:** Significant differences between patients with CAP and HCAP were tested using chi-square, or Fisher’s exact test. Data are mean (SD), number (%) patients, or median (range).

**Abbreviations:** BUN, blood urea nitrogen; BNP, brain natriuretic peptide; BMI, body mass index; CAP, community-acquired pneumonia; COPD, chronic obstructive pulmonary disease; CRP, C-reactive protein; ECOG, eastern cooperative oncology group; HCAP, healthcare-associated pneumonia; ICU, intensive care unit; qSOFA, quick sequential organ failure assessment

### Viral profiles in pneumonia

Among the 452 enrolled patients, 430 (95.1%) patients were tested for respiratory viruses in sputum or endotracheal aspirates. The remaining 22 (4.9%) patients underwent RT-PCR on nasopharyngeal specimens. Additionally, BAL fluid RT-PCR was performed in 16 (3.5%) patients, and 4 (0.9%) patients underwent pleural effusion RT-PCR. Fifty-four (23.7%) of the patients with HCAP were found to have a viral infection; 31 (13.8%) patients had a pure viral infection and 23 (10.3%) patients had a viral–bacterial coinfection. In the CAP group, 74 (32.5%) patients had a viral infection, and the viral infection rate and viral–bacterial coinfection rates were 24.6% and 7.9% respectively. The viral infection rate was significantly lower in the HCAP patients than in the CAP patients, irrespective of whether viral–bacterial infections were included (viral infection with viral–bacterial coinfection, p = 0.049, viral infection only, p = 0.004). The respiratory viruses identified are summarized in Table 2. In both HCAP and CAP groups, influenza A was the most common respiratory virus, followed by entero-rhinovirus. The general viral profile of patients with HCAP and CAP were similar, except for influenza A and adenovirus, which were higher in the CAP group than the HCAP (p = 0.040 and p = 0.001, respectively). Among the 16 patients who underwent BAL fluid RT-PCR, 14 (87.5%) patients demonstrated the same results as those obtained from sputum RT-PCR. In the remaining 2 patients, no virus was identified by sputum RT-PCR, but respiratory syncytial virus and influenza A were identified, respectively, in BAL fluid RT-PCR. One of 4 patients had a positive viral result on pleural effusion RT-PCR and this was identical to the endotracheal aspirate RT-PCR. The viral profiles, according to respiratory specimens, are shown in S2 Table.

**Table 2:** Profile of pathogens identified in patients with HCAP or CAP.

	Total (n = 235)	HCAP (n = 134)	CAP (n = 101)	*P* value
**Viral**				
Influenza virus A	44 (18.7)	19 (14.2)	25 (24.8)	0.040
Entero-Rhinovirus	29 (12.3)	15 (11.2)	14 (13.9)	0.538
Human metapneumovirus	14 (6.0)	5 (3.7)	9 (8.9)	0.097
Respiratory syncytial virus	14 (6.0)	5 (3.7)	9 (8.9)	0.097
Parainfluenza virus	11 (4.7)	7 (5.2)	4 (4.0)	0.650
Parainfluenza virus 1	4 (1.7)	2 (1.5)	2 (2.0)	1.000
Parainfluenza virus 2	2 (0.9)	2 (1.5)	0 (0.0)	0.508
Parainfluenza virus 3	5 (2.1)	3 (2.2)	2 (2.0)	1.000
Adenovirus	8 (3.4)	0 (0.0)	8 (7.9)	0.001
Coronavirus	7 (3.0)	5 (3.7)	2 (2.0)	0.702
Coronavirus 229E	1 (0.4)	1 (0.7)	0 (0.0)	1.000
Coronavirus NL63	1 (0.4)	0 (0.0)	1 (1.0)	0.430
+Coronavirus OC43	5 (2.1)	4 (3.0)	1 (1.0)	0.394
Influenza virus B	5 (2.1)	1 (0.7)	4 (4.0)	0.168
**Bacterial**				
Multi-bacterial	27 (11.5)	21 (15.7)	6 (5.9)	0.021
Multidrug-resistant	39 (16.6)	35 (26.1)	4 (4.0)	< 0.001
*Streptococcus pneumoniae*	40 (17.0)	22 (16.4)	18 (17.8)	0.777
*Klebsiella pneumoniae*	32 (13.6)	22 (16.4)	10 (9.9)	0.149
*Pseudomonas aeruginosa*	24 (10.2)	20 (14.9)	4 (4.0)	0.006
MRSA	21 (8.9)	18 (13.4)	3 (3.0)	0.005
MSSA	16 (6.8)	8 (6.0)	8 (7.9)	0.557
*Escherichia coli*	12 (5.1)	10 (7.5)	2 (2.0)	0.059
*Haemophilus influenzae*	8 (3.4)	7 (5.2)	1 (1.0)	0.142
*Enterobacter cloacae*	6 (2.6)	3 (2.2)	3 (3.0)	1.000
*Acinetobacter baumannii*	6 (2.6)	5 (3.7)	1 (1.0)	0.241
*Serratia marcescens*	3 (1.3)	3 (2.2)	0 (0.0)	0.553
*Mycoplasma pneumoniae*	2 (0.9)	0 (0.0)	2 (2.0)	0.103
*Stenotrophomonas* *maltophilia*	2 (0.9)	2 (1.5)	0 (0.0)	0.218
*Citrobacter freundii*	1 (0.4)	1 (0.7)	0 (0.0)	1.000
**Others**				
*Pneumocystis jirovecii*	2 (0.9)	2 (1.5)	0 (0.0)	0.508

**Note:** Significant differences between patients with CAP and HCAP were tested using chi-square, or Fisher’s exact test. Data are mean (SD), number (%) patients, or median (range).

**Abbreviations:** CAP, community-acquired pneumonia; HCAP, healthcare-associated pneumonia; MRSA, Methicillin-resistant *Staphylococcus aureus*; MSSA, Methicillin-sensitive *Staphylococcus aureus*

### Bacterial profiles in pneumonia

More than half of the patients (64.9%) with HCAP received antibiotics at the healthcare facilities and 21 (20.8%) patients with CAP received any form of antibiotics prior to our hospital. In contrast to viral infection, the proportion of bacterial pneumonia was significantly higher in patients with HCAP than in those with CAP (35.7% vs 11.8%, p < 0.001). Table 2 shows the bacterial pathogens in patients with HCAP or CAP. *Pseudomonas aeruginosa* and methicillin-resistant *Staphylococcus aureus* (MRSA) were more frequently observed in HCAP than in CAP patients (p = 0.006 and p = 0.005, respectively). The proportion of multi-bacterial and multidrug-resistant (MDR) bacterial infection was also higher in the HCAP than CAP group (p = 0.021 and p < 0.001, respectively). Empirical antimicrobial treatment regimens for HCAP or CAP are summarized in S3 Table.

### Seasonal distribution of pneumonia

**Fig 2:**
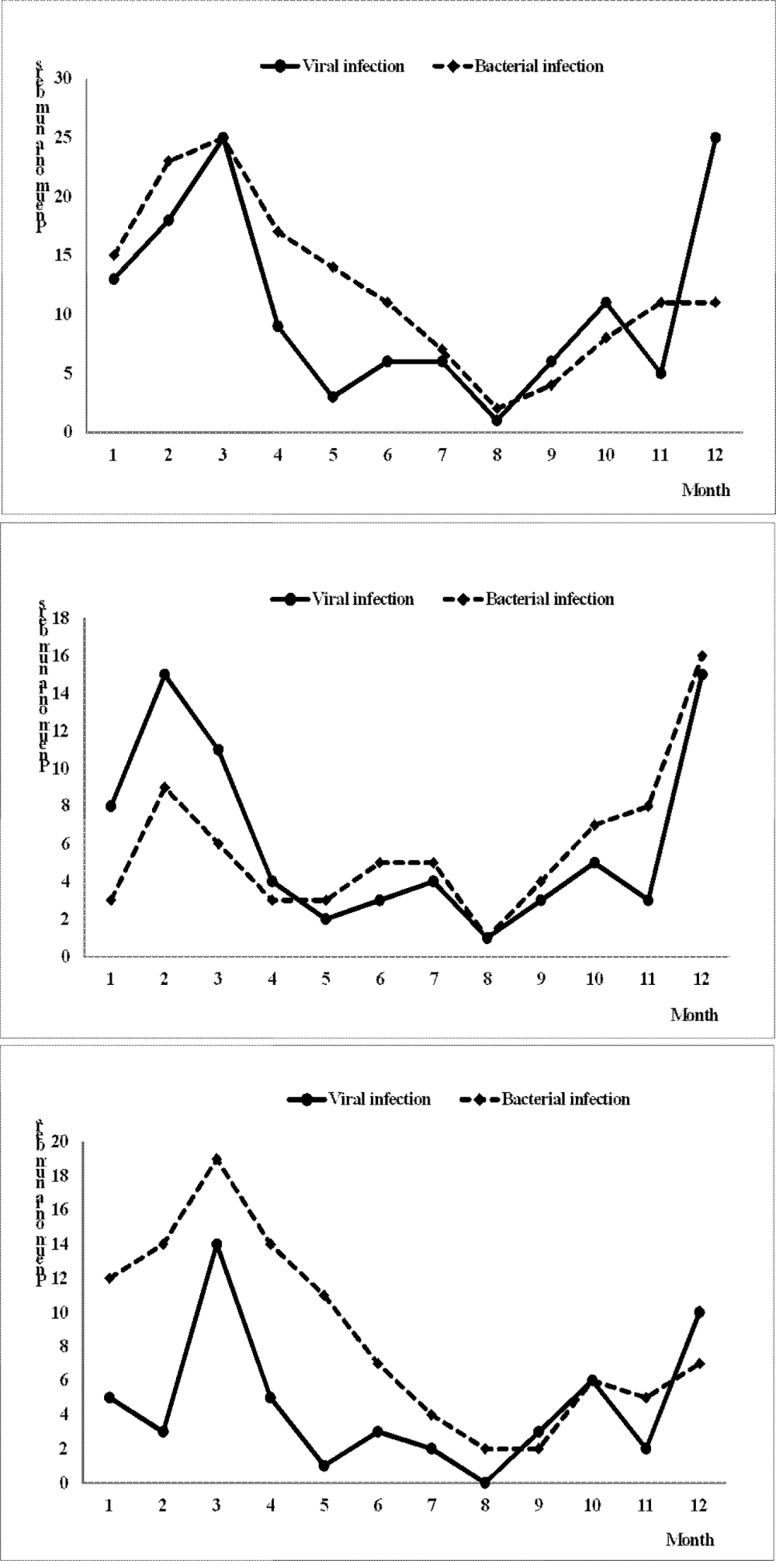
Monthly distribution of respiratory viral or bacterial pneumonia. The x-axis shows each month and the y-axis shows the number of observed pneumonia events. The number of viral and bacterial pneumonia events in each month. (upper panel: all CAP and HCAP patients, middle panel: CAP patients only; and lower panel: HCAP patients only).

**Fig 3:**
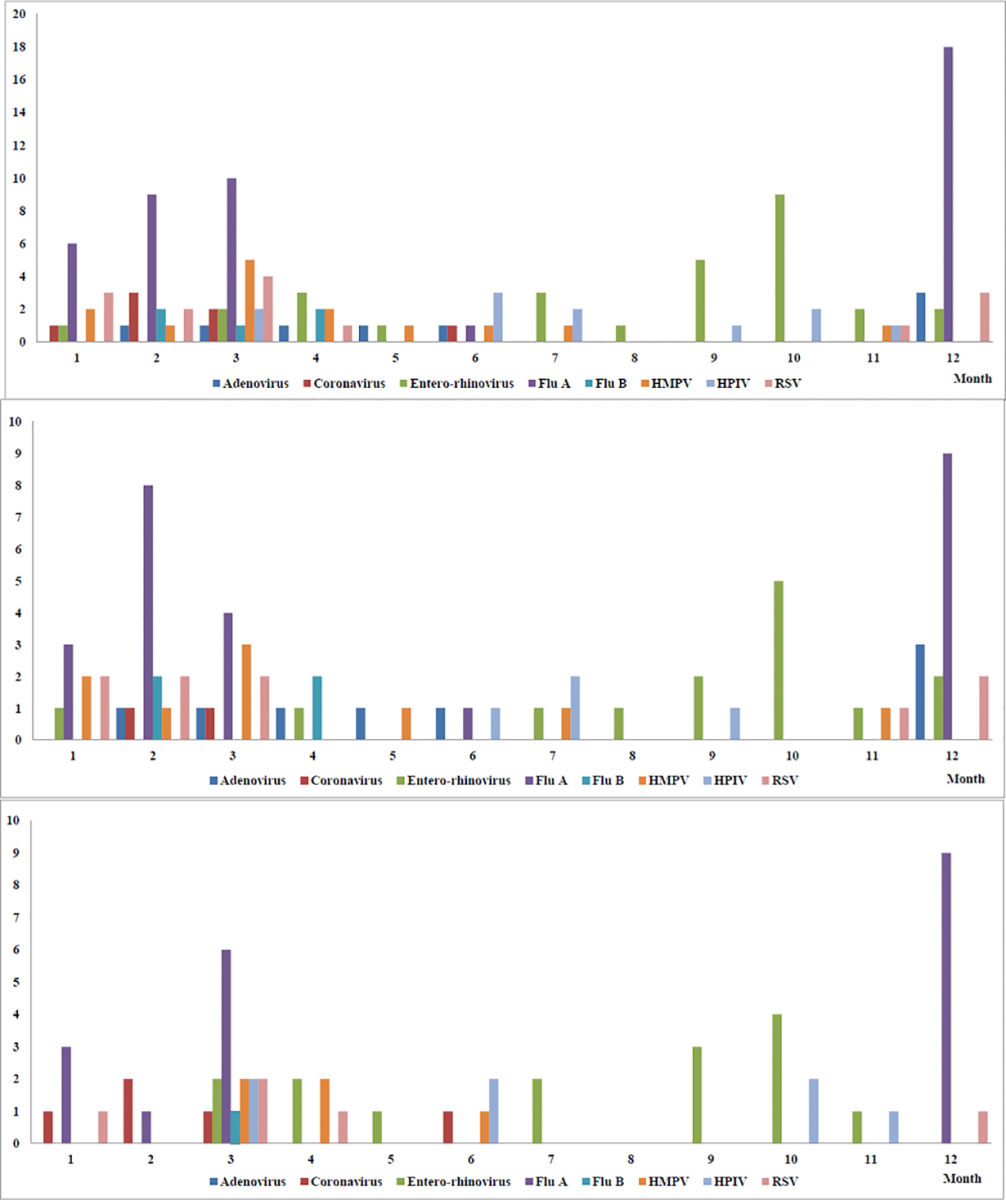
Monthly distribution of each respiratory virus. The x-axis shows each month and the y-axis shows the number of observed pneumonia events. (upper panel: all CAP and HCAP patients, middle panel: CAP patients only; and lower panel: HCAP patients only).

Fig 2 shows the monthly distribution of viral and bacterial infection. In the HCAP group, bacterial infections were predominant during all 4 seasons, and were more common from February to April. The seasonal variation curve of viral infections was similar to that in the CAP group, but with a tendency to lag 1 month behind that in the CAP group. Fig 3 shows the distribution of each virus. Generally, the viral seasonal distribution in the HCAP group was similar to that in the CAP group, except for adenovirus and human metapneumovirus. Of the 44 patients with influenza pneumonia, more than half (63.6%) had a history of influenza vaccination.

### Outcomes of pneumonia and predictors of mortality

The median length of hospital stay (LOS) was 8.2 (range: 0.0–120.0) days. Patients with HCAP had a significantly longer LOS than did patients with CAP (7.1 *vs*. 9.8 days, p = 0.009). Thirty-one (6.9%) patients died in the hospital; the HCAP group had a higher mortality than did the CAP group (6.7% *vs*. 3.5%, p = 0.004). After hospitalization, 344 (76.15) returned home and 76 (16.8%) patients were transferred to a nursing care home. When each group was considered separately, the CAP patients with viral infection, or viral–bacterial coinfection, or bacterial infection had a similar hospital LOS (p = 0.330), in-hospital mortality (p = 0.269), and transfer rate to a nursing home (p = 0.191). Likewise, viral infection did not affect the clinical prognosis in the HCAP group, including the hospital LOS (p = 0.377), mortality (p = 0.930), and discharge rate to a nursing home (p = 0.184). Multivariate analysis revealed that MRSA infection (OR 8.79, 95% CI 1.474–52.360, p = 0.017), BUN (OR 1.04, 95% CI 1.012–1.072, p = 0.006), and lactic acid (OR 1.18, 95% CI 1.012–1.372, p = 0.034) were independent predictors of in-hospital mortality in patients with HCAP or CAP. Moreover, MRSA infection was the strongest predictor of in-hospital mortality in HCAP patients (OR 10.0, 95% CI 1.331–75.619, p = 0.025).

## Discussion

In this study, we investigated the microbiological profile of patients with HCAP, focusing on viral infections. To date, only limited studies have evaluated the viral profile of patients with HCAP [11, 31, 32]. However, these studies only included patients with severe pneumonia requiring ICU care focusing on the CAP, which involved only a limited number of HCAP patients [32]. Furthermore, they did not strictly control the respiratory sampling time and did not specified whether the patients received antibiotics and how long they received the antibiotics prior to admission [31]. Lastly, only limited respiratory specimens were used for detection of respiratory viruses, mainly nasopharyngeal swab [31, 32]. Therefore, the general trend and microbiological profile in HCAP remained unclear. In the present study, we evaluated the role of respiratory viruses in patients with mild to severe HCAP, in comparison to that in patients with CAP, using all available respiratory specimens including sputum or endotracheal aspirate, BAL fluid or even pleural effusion. Unlike previous studies [31], microbiological results showed some differences in both groups. The overall viral infection rates were lower in the HCAP group (23.7%) than in the CAP group (32.5%). Most importantly, influenza A viral infection was significantly lower in the patients with HCAP than those with CAP (p = 0.040). There are several plausible explanations for this. In the previous multicenter study, bronchial asthma [odds ratio (OR) 4.006], male gender (OR 3.507) and age ≥ 50 years (OR 2.653) were the independent risk factors for influenza A viral pneumonia [33]. Moreover, chronic lung diseases, such as bronchial asthma and chronic obstructive pulmonary disease, are well-known risk factors for viral infections [34, 35]. In this study, the proportion of male was similar and the patients with CAP were younger. Therefore, male gender and age might not be the reason. Patients with CAP had a higher prevalence of chronic lung disease than did patients with HCAP. Therefore, the CAP group may have had a higher chance of infection by respiratory viruses, especially influenza. Furthermore, more than half of the HCAP patients (58.9%) were hospitalized for 2 or more days in the 90 days before pneumonia. Therefore, they may have had little opportunity of coming into contact with other patients who carried respiratory viruses [11]. Similar to previous studies [14, 17, 22, 36], influenza A was the most commonly identified respiratory virus in CAP patients, and this was also true for the HCAP group. Interestingly, more than half of the patients (63.6%) had a history of influenza vaccination. Among them, influenza subtype H3N2 was detected in 5 (11.4%) patients and influenza A subtype H1N1 was found in 39 (88.6%) patients. Most of the participants were elderly patients with mean age 70.5 years. Several studies showed that vaccine efficacy against influenza was decreased with increasing age although there were several confounding factors [37, 38]. Since there was no available data for vaccine constituents, it is difficult to draw conclusions about the efficacy of influenza vaccine in this study. Further studies are required to determine the relationship between the effectiveness of influenza vaccines and age.

In this study, viral pneumonia itself was not found to affect the clinical prognosis, such as LOS and in-hospital mortality in either group. Recent studies of CAP have shown similar results [22, 31], and we further showed that viral infection had no significant association with increased mortality in patients with HCAP. Contradictory to the previous studies that reported viral–bacterial coinfection as an important risk factor for mortality in the CAP [22, 32], no significant difference was noted in this study. Most previous studies obtained samples for PCR via a nasopharyngeal or oropharyngeal swab [22, 39], but this may lead to false results in cases of viral pneumonia [11, 23]. Easier access to a tertiary referral hospital, due to the healthcare system in Korea [40], might be another reason for the similar prognosis of patients with viral–bacterial coinfection, and viral and bacterial infections. One thing we have to mention here is that the results of this study should be interpreted with caution, because the true bacterial profile of these patients is not known. In the present study, we enrolled only the patients who did not receive antibiotics for more than 24 hrs prior to our hospital to increase the yield of microbiological test. Moreover, the conventional microbiological studies, including blood culturing and sputum or endotracheal aspirate Gram staining and culturing, were conducted before or within 1 hr after antibiotics administration. However, we obtained low rates of bacterial detection similar to the previous studies [41–43]: bacterial pathogens were identified in 35.7% of patients with HCAP and in 11.8% of patients with CAP. This could be the frailty of Gram staining and culture for bacterial detection. There is a report that the use of multi-bacterial molecular testing approach approximately doubles pathogen detection in patients with pneumonia [44]. Particularly, they reported that PCR detected significantly more *Haemophilus influenzae*, *Streptococcus pneumoniae*, *Moraxella catarrhalis*, *Staphylococcus aureus*, *Escherichia coli*, and *Klebsiella pneumoniae* than standard culture-based methods [44]. In this study, only 63 (26.8%) patients (24 patients with HCAP and 39 patients with CAP) were conducted PCR for *Mycoplasma pneumoniae*, and two patients with CAP showed positive results. We conducted this study according to our usual clinical practice and multi-bacterial PCR is currently not available in our hospital. However, more timely and sensitive microbiological methods like PCR might be necessary to enable early bacterial detection and pathogen-directed therapy.

In the present study, MRSA infection was the strongest predictor of in-hospital mortality, especially in HCAP patients (OR 10.0, 95% CI 1.331–75.619, p = 0.025) after adjusting for age, sex, BMI, smoking history, ECOG status and comorbidities. Actually, it is well known that MRSA pneumonia results in numerous complications and high mortality rates [45]. Furthermore, residency in a long-term care facility is identified as a risk factor for MRSA rather than hospitalization or prescription of antimicrobials in hospitals in the recent studies [46]. We have confirmed that fact again in this study.

HCAP was removed from the updated 2016 HAP/VAP guidelines [10], because many studies have reported that HCAP patients were not infected with MDR pathogens [6–9]. However, in the present study, the HCAP patients showed significantly higher MDR bacterial infection rates. We performed a multivariate analysis adjusting for age, sex, BMI, performance status (ECOG ≥ 3), comorbidities, and pneumonia severity indexes (PSI, CURB 65 ≥ 3, qSOFA ≥ 2), and found that HCAP is the most important predictor for MDROs (OR 14.2, 95% CI 1.734–115.9, p = 0.013) unlike previous studies [6–9]. Recently, the number of elderly patients with frequent hospital contacts has increased rapidly and many of these individuals are residing in nursing homes or long-term care facilities, especially in Korea [47], and those HCAP patients might be prone to MDR bacterial infection [48–50]. Such recent trends may explain why this study has shown results that are different from previous studies. However, further studies with updated data are needed to confirm these findings.

Our study has several limitations. First, a limited number of invasive respiratory specimens was available in this study. However, BAL is not a preferred technique, because of its invasiveness [3, 10], and good concordance has been found between the results of cultures of sputum and transtracheal aspirates, particularly when good–quality sputum specimens are obtained [27, 51, 52]. In this study, Gram staining and culturing of the expectorated sputum produced a relatively high yield, and sputum RT-PCR showed a similar effectiveness as the BAL fluid RT-PCR (87.5%). Second, we only included admitted patients with HCAP or CAP, and pneumonia patients in an outpatient setting were not included. Previous studies that included outpatients reported that the viral pneumonia rate was as high as 36% [13], and there may be a risk that our results underestimated the proportion of viral pneumonia. Lastly, this study was conducted in only one tertiary referral hospital and thus may reflect this limited clinical context. A prospective multicenter study of HCAP and CAP is needed to confirm out findings.

## Conclusion

HCAP patients have a lower likelihood of viral infection than, and a similar viral profile and seasonal variations to patients with CAP. Viral infection did not affect the prognosis of patients with HCAP. Rather, multi-bacterial or MDR bacterial infection was the most important concern in patients with HCAP.

## Supporting information

S1 Table Demographic findings in patients with viral or bacterial pneumonia. (DOC)

S2 Table Viral profile according to respiratory specimens. (DOC)

S3 Table Empirical antimicrobial treatment regimens in the patients with HCAP or CAP. (DOC)

## Abbreviations

BAL: bronchoalveolar lavage
BMI: body mass index
CAP: community-acquired pneumonia
ECOG: eastern cooperative oncology group
HAP: hospital-acquired pneumonia
HCAP: healthcare-associated pneumonia
ICU: intensive care unit
LOS: length of hospital stay
MDR: multidrug-resistant
MRSA: methicillin-resistant *Staphylococcus aureus*
PCR: polymerase chain reaction
PSI: pneumonia severity index
qSOFA: quick sepsis-related organ failure assessment
RT-PCR: reverse-transcription polymerase chain reaction
SD: standard deviation
VAP: ventilator-associated pneumonia
