# Supplementary material for: Comparison of viral infection in healthcare-associated pneumonia (HCAP) and community-acquired pneumonia (CAP)
Source: PLoS One. 2018 Feb 15;13(2):e0192893. doi: 10.1371/journal.pone.0192893 (PMC5813982; doi:10.1371/journal.pone.0192893)
Supplement: S1 Table — (DOC) [file pone.0192893.s001.doc]

S1 Table. Demographic findings in patients with viral or bacterial pneumonia

|  | Viral (n=87) | Viral and bacterial (n = 41) | Bacterial (n= 107) | *P* value |
| --- | --- | --- | --- | --- |
| Male sex | 40 (46.0) | 25 (61.0) | 79 (73.8) | < 0.001 |
| Age, years | 68.1 (17.6) | 70.5 (15.1) | 73.3 (12.0) | 0.057 |
| BMI, kg/m^2^ | 22.2 (4.1) | 21.9 (4.3) | 20.6 (4.5) | 0.036 |
| Ever-smoker | 19 (21.8) | 11 (26.8) | 32 (29.9) | 0.654 |
| ECOG ≥ 3 | 32 (36.8) | 21 (51.2) | 66 (61.7) | 0.002 |
| Comorbidities |  |  |  |  |
| Diabetes mellitus | 23 (26.4) | 13 (31.7) | 35 (32.7) | 0.626 |
| Hypertension | 40 (46.0) | 17 (41.5) | 59 (55.1) | 0.243 |
| Chronic lung disease | 29 (33.3) | 7 (17.1) | 23 (21.5) | 0.072 |
| Bronchiectasis | 8 (9.2) | 1 (2.4) | 5 (4.7) | 0.243 |
| COPD | 13 (14.9) | 5 (12.2) | 16 (15.0) | 0.903 |
| Bronchial asthma | 13 (14.9) | 4 (9.8) | 4 (3.7) | 0.024 |
| Interstitial lung disease | 0 (0.0) | 0 (0.0) | 2 (1.9) | 0.302 |
| Aspiration pneumonia | 7 (8.0) | 10 (24.4) | 42 (39.3) | < 0.001 |
| Pneumonia severity index | 95.2 (39.0) | 109.2 (35.1) | 120.6 (31.9) | < 0.001 |
| CURB-65 ≥ 2 | 38 (43.7) | 26 (63.4) | 70 (65.4) | 0.006 |
| CURB-65 ≥ 3 | 11 (12.6) | 12 (29.3) | 26 (24.3) | 0.048 |
| qSOFA ≥ 2 | 6 (6.9) | 9 (22.0) | 27 (25.2) | 0.003 |
| Direct sub-ICU admission | 3 (3.4) | 2 (4.9) | 19 (17.8) | 0.002 |
| Direct ICU admission | 4 (4.6) | 4 (9.8) | 7 (6.5) | 0.539 |

**Note:** Significant differences between patients with viral infection, viral-bacterial infection, and bacterial infection were tested using ANOVA or Fisher’s exact test. Data are mean (SD), number (%) patients, or median (range).

**Abbreviations:** BMI, body mass index; CAP, community-acquired pneumonia; COPD, chronic obstructive pulmonary disease; ECOG, eastern cooperative oncology group; HCAP, healthcare-associated pneumonia; ICU, intensive care unit; qSOFA, quick sequential organ failure assessment;
