# Supplementary material for: Comparison of viral infection in healthcare-associated pneumonia (HCAP) and community-acquired pneumonia (CAP)
Source: PLoS One. 2018 Feb 15;13(2):e0192893. doi: 10.1371/journal.pone.0192893 (PMC5813982; doi:10.1371/journal.pone.0192893)
Supplement: S2 Table — (DOC) [file pone.0192893.s002.doc]

S2 Table. Viral profile according to respiratory specimens

|  | Total  (n = 452) | HCAP  (n = 224) | CAP  (n = 228) | P value |
| --- | --- | --- | --- | --- |
| **Viral multiplex RT-PCR noninvasive specimen** |  |  |  | 0.693 |
| Any respiratory virus | 128 (28.3) | 54 (24.1) | 74 (32.5) | 0.049 |
| Adenovirus | 7 (1.5) | 0 (0.0) | 7 (3.1) | 0.015 |
| Coronavirus | 7 (1.5) | 5 (2.2) | 2 (0.9) | 0.281 |
| Coronavirus 229E | 1 | 1 | 0 | 0.496 |
| Coronavirus NL63 | 1 | 0 | 1 | 1.000 |
| Coronavirus OC43 | 5 | 4 | 1 | 0.213 |
| Entero-Rhinovirus | 30 (6.6) | 16 (7.1) | 14 (6.1) | 0.669 |
| Influenza virus A | 43 (9.5) | 19 (8.5) | 24 (10.5) | 0.459 |
| Influenza virus B | 5 (1.1) | 1 (0.4) | 4 (1.8) | 0.372 |
| Human metapneumovirus | 15 (3.3) | 5 (2.2) | 10 (4.4) | 0.201 |
| Parainfluenza virus | 11 (2.4) | 7 (3.1) | 4 (1.8) | 0.344 |
| Parainfluenza virus 1 | 4 | 2 | 2 | 1.000 |
| Parainfluenza virus 2 | 2 | 2 | 0 | 0.245 |
| Parainfluenza virus 3 | 5 | 3 | 2 | 0.683 |
| Respiratory syncytial virus | 13 (2.9) | 4 (1.8) | 9 (3.9) | 0.169 |
| *Sputum or endotracheal aspirate* | n = 430 | n = 214 | n = 216 |  |
| Any viruses | 124 (28.8) | 52 (24.3) | 72 (33.3) | 0.039 |
| Adenovirus | 7 (1.6) | 0 (0.0) | 7 (3.2) | 0.015 |
| Coronavirus | 7 (1.6) | 5 (2.3) | 2 (0.9) | 0.283 |
| Entero-Rhinovirus | 27 (6.3) | 14 (6.5) | 13 (6.0) | 0.823 |
| Influenza virus A | 42 (9.8) | 19 (8.9) | 23 (10.6) | 0.537 |
| Influenza virus B | 5 (1.2) | 1 (0.5) | 4 (1.9) | 0.372 |
| Human metapneumovirus | 15 (3.5) | 5 (2.3) | 10 (4.6) | 0.195 |
| Parainfluenza virus | 11 (2.6) | 7 (3.3) | 4 (1.9) | 0.351 |
| Respiratory syncytial virus | 13 (3.0) | 4 (1.9) | 9 (4.2) | 0.164 |
| *Nasopharyngeal swab* | n = 22 | n = 10 | n = 12 |  |
| Any viruses | 4 (18.2) | 2 (20.0) | 2 (16.7) | 1.000 |
| Entero-Rhinovirus | 3 (13.6) | 2 (20.0) | 1 (8.3) | 0.571 |
| Influenza virus A | 1 (4.5) | 0 (0.0) | 1 (8.3) | 1.000 |
| **Viral multiplex RT-PCR invasive specimen** |  |  |  |  |
| *BAL or deep bronchial washing* | n = 16 | n = 10 | n= 6 |  |
| Any viruses | 6 (37.5) | 3 (30.0) | 3 (50.0) | 0.607 |
| Coronavirus OC43 | 2 (12.5) | 2 (20.0) | 0 (0.0) | 0.500 |
| Influenza virus A | 1 (6.3) | 0 (0.0) | 1 (16.7) | 0.375 |
| Influenza virus B | 1 (6.3) | 0 (0.0) | 1 (16.7) | 0.375 |
| Respiratory syncytial virus | 2 (12.5) | 1 (10.0) | 1 (16.7) | 1.000 |
| *Pleural effusion* | n = 4 | n = 0 | n = 4 |  |
| Adenovirus | 1 (25.0) | 0 (0.0) | 1 (25.0) |  |

**Note:** Significant differences between patients with CAP and HCAP were tested using chi-square, or Fisher’s exact test. Data are mean (SD), number (%) patients, or median (range).

**Abbreviations:** CAP, community-acquired pneumonia; HCAP, healthcare-associated pneumonia
