# Supplementary material for: Comparison of viral infection in healthcare-associated pneumonia (HCAP) and community-acquired pneumonia (CAP)
Source: PLoS One. 2018 Feb 15;13(2):e0192893. doi: 10.1371/journal.pone.0192893 (PMC5813982; doi:10.1371/journal.pone.0192893)
Supplement: S3 Table — (DOC) [file pone.0192893.s003.doc]

S3 Table. Empirical antimicrobial treatment regimens in the patients with HCAP or CAP

|  | HCAP  (n = 224) | CAP  (n = 228) |
| --- | --- | --- |
| 3^rd^ cephalosporin | 3 (1.3) | 11 (4.9) |
| 3^rd^ cephalosporin + azithromycin | 32 (14.3) | 118 (52.2) |
| 3^rd^ cephalosporin + fluoroquinolone | 33 (14.7) | 38 (16.8) |
| 3^rd^ cephalosporin + clindamycin | 13 (5.8) | 9 (4.0) |
| Fluoroquinolone | 23 (10.3) | 16 (7.1) |
| Fluoroquinolone + bactrim | 3 (1.3) | 0 (0.0) |
| Ampicillin/sulbactam | 0 (0.0) | 2 (0.9) |
| Piperacillin/tazobactam | 78 (34.8) | 19 (8.4) |
| Piperacillin/tazobactam + fluoroquinolone | 26 (11.6) | 12 (5.3) |
| Piperacillin/tazobactam + amikacin | 0 (0.0) | 1 (0.4) |
| Piperacillin/tazobactam + bactrim | 1 (0.4) | 0 (0.0) |
| Piperacillin/tazobactam + vancomycin | 3 (1.3) | 0 (0.0) |
| Carbapenem | 6 (2.7) | 0 (0.0) |
| Carbapenem + vancomycin | 1 (0.4) | 0 (0.0) |
| Vancomycin + colistin | 1 (0.4) | 0 (0.0) |

**Note:** Data are number (%) patients.

**Abbreviations:** CAP, community-acquired pneumonia; HCAP, healthcare-associated pneumonia
